# Role of the lipoxin A4 receptor in the development of neutrophil extracellular traps in *Leishmania infantum* infection

**DOI:** 10.1186/s13071-019-3530-8

**Published:** 2019-05-29

**Authors:** Furong Wei, Wenci Gong, Junyun Wang, Yuetao Yang, Jianxiu Liu, Yanjuan Wang, Jianping Cao

**Affiliations:** 1 National Institute of Parasitic Diseases, Chinese Center for Disease Control and Prevention, Shanghai, 200025 China; 2 Chinese Center for Tropical Diseases Research, Shanghai, 200025 China; 3 WHO Collaborating Center for Tropical Diseases, Shanghai, 200025 China; 4 National Center for International Research on Tropical Diseases, Ministry of Science and Technology, Shanghai, 200025 China; 5 Key Laboratory of Parasite and Vector Biology, Ministry of Health, Shanghai, 200025 China

**Keywords:** Lipoxin A4 receptor, *Leishmania infantum*, Citrullinated histone H3, Neutrophil extracellular traps

## Abstract

**Background:**

Neutrophils play an immunomodulatory role through the release of neutrophil extracellular traps (NETs). NETs are released in response to *Leishmania* infection, but the mechanism of NET extrusion has not been elucidated. The lipoxin A4 receptor on neutrophils is crucial for the inflammatory response and immune regulation of many diseases, including *Leishmania* infection. Therefore, in the present study, we tried to explore whether *Leishmania infantum* promastigotes stimulate neutrophil activation and NET release *via* activating the lipoxin A4 receptor.

**Results:**

*Leishmania infantum* promastigotes stimulated neutrophil activity, but blocking of the lipoxin A4 receptor with its antagonist Boc prior to *L. infantum* stimulation abrogated these effects. Neutrophils showed citrullinated histone H3 expression and simultaneous NET extrusion on *L. infantum* stimulation, but a decline in both was observed on blocking of the lipoxin A4 receptor. Moreover, differentiated HL-60 cells with lipoxin A4 receptor silencing showed a decrease in citrullinated histone H3 expression as compared to the unsilenced HL-60 samples on stimulation with promastigotes.

**Conclusions:**

*Leishmania infantum* promastigotes altered the characteristics of neutrophils and induced NET extrusion by activating the lipoxin A4 receptor. The lipoxin A4 receptor may have potential as a therapeutic target in relation to NET extrusion in the treatment of leishmaniasis, but its mechanisms of action need to be explored in more depth.

## Background

Leishmaniasis, a vector-borne disease caused by a protozoan flagellate of the genus *Leishmania* and transmitted by female phlebotomine sand flies, causes a group of diseases which have symptoms that differ according to the causative *Leishmania* species and the host’s ability to develop and control immune responses [1]. The adaptive immune response is known to be critical for resolving *Leishmania* infection, but innate immune mechanisms have been receiving much attention in the context of this disease, especially the activity of neutrophils as anti-microbial effector molecules [2–4].

Neutrophils, as the largest group of immune cells, recruited by numerous parasite-, host- and sand fly-derived chemotactic factors in the process [4], are the first to reach the *Leishmania* infiltration site [5–8] and internalize the parasite [7, 9]. The internalization process is tightly regulated through three major strategies: phagocytosis, degranulation and the release of neutrophil extracellular traps (NETs). The process of NET release involves the decondensation of chromatin and the disruption of nuclear and granular architecture to allow the mixing of chromatin and antimicrobial granular content in the cytoplasm; this subsequently leads to the release of the chromatin network with anchored granular proteins, such as elastase and myeloperoxidase (MPO), into the extracellular space [10]. This novel type of cell program is called NETosis, and NETs are primarily comprised of DNA backbone fibers, antibacterial proteins/peptides, granule proteins, etc.

Neutrophils release NETs in response to *L. amazonensis*, *L. infantum*, *L. major*, *L. donovani* and *L. mexicana* infection [9, 11–13]. Some *Leishmania* species can be captured and killed by NETs [11], while some are protected from NET-mediated killing by lipophosphoglycan [13], the enzyme 3ʹ-nucleotidase/nuclease [12] or by other factors that have yet to be identified [9]. Although many aspects of the immune response to *Leishmania* species have been unveiled, little is known in terms of the mechanisms of activation of NETosis and the role of NETs.

*Leishmania* promastigotes, by releasing a chemokine called *Leishmania* chemotactic factor, can recruit and activate neutrophils *via* the lipoxin A4 receptor [14]. The lipoxin A4 receptor is crucial for inflammatory response and immune regulation in many diseases [15–17]. In particular, many studies have focused on the role of the lipoxin A4 receptor in neutrophil activation [18]. It has been demonstrated that, in addition to regulation of neutrophil activation and recruitment, this receptor has a profound influence on neutrophil survival and apoptosis with contrasting effects, by mediating aggravation or resolution of the inflammatory response [19]. In particular, anti-inflammatory lipoxin A4 receptor signaling induced by *Leishmania* promastigotes has been found to increase the phagocytotic ability and enable intracellular survival of *Leishmania* parasites [20]. Given these complex roles of the lipoxin A4 receptor in neutrophil activity, we were interested in investigating whether *Leishmania* promastigotes induce NETosis by activating the lipoxin A4 receptor. This study is based on the hypothesis that the lipoxin A4 receptor plays an important role in determining neutrophil fate following neutrophil activation *via* mediating the production on NETs. This receptor may therefore provide a new pharmacological target in the process of NETosis that occurs during parasitic infections.

## Results

### *Leishmania infantum* promastigotes induce the release of NETs and are trapped by NETs

**Fig. 1:**
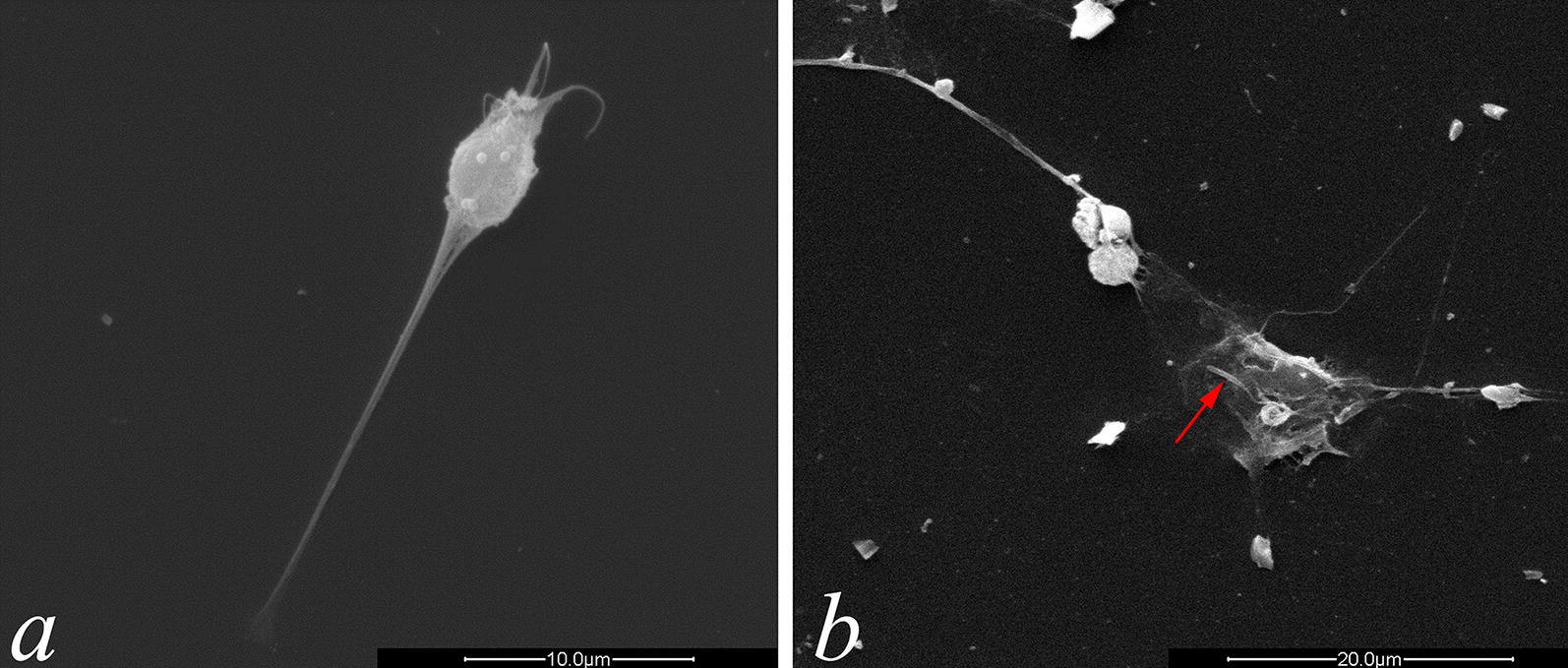
Scanning electron microscope images of the neutrophils stimulated by *L. infantum* promastigotes for 5 h. Purified neutrophils were exposed to *L. infantum* promastigotes for 5 h. The coverslips seeded with samples were fixed with 1 ml of Monti-Graziadei solution and processed for scanning electron microscopy to visualize NET release. **a** NET structure of a single neutrophil. **b** NET threads within which a promastigote is trapped (red arrow). *Scale-bars*: **a**, 10 µm; **b**, 20 µm

To confirm the potential of *L. infantum* to induce the formation of NETs, we cocultured *L. infantum* promastigotes with neutrophils for 5 h. The neutrophils lost their typical rounded morphology and released NETs, which appeared as filamentous structures under a scanning electron microscope (Fig. 1a). Interestingly, the *L. infantum* promastigotes were found to be entrapped in the NET fibers (Fig. 1b).

### The lipoxin A4 receptor is associated with the expression of citrullinated histone H3 during *L. infantum* stimulation

**Fig. 2:**
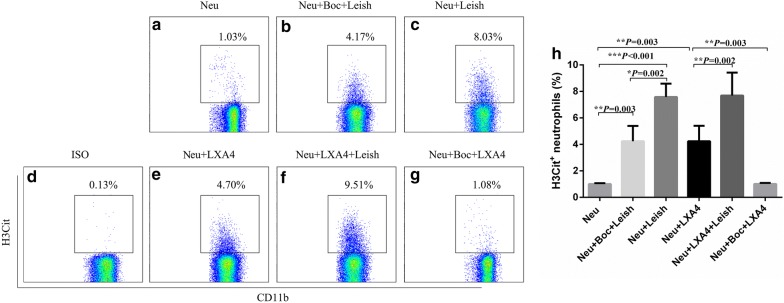
Flow cytometry analysis of H3Cit formation in neutrophils from whole blood stimulated by *L. infantum* promastigotes for 5 h. Whole blood pre-treated with or without the antagonist (Boc) or the agonist lipoxin A4 (LXA4) were exposed to *L. infantum* promastigotes or left untreated for 5 h at 37 °C. Percentage of H3Cit formation in neutrophils was quantified by flow cytometric assay. **a** Percentage of H3Cit-positive neutrophils in experimental group Neu; **b** Percentage of H3Cit-positive neutrophils in experimental group Neu+Boc+Leish; **c** Percentage of H3Cit-positive neutrophils in experimental group Neu+Leish; **d** Isotype of H3Cit; **e** Percentage of H3Cit-positive neutrophils in experimental group Neu+LXA4; **f** Percentage of H3Cit-positive neutrophils in experimental group Neu+LXA4+Leish; **g** Percentage of H3Cit-positive neutrophils in experimental group Neu+Boc+LXA4; **h** Statistical analysis of the H3Cit-positive neutrophil percentages among experimental groups. The differences were analyzed using one-way ANOVA, then LSD was applied in the following two groups’ comparison. *P < 0.05, **P < 0.01, ***P < 0.001. *Abbreviations*: Neu: control group of untreated whole blood; Neu+Boc+Leish, Boc-pretreated whole blood stimulated by *L. infantum* promastigotes; Neu+Leish, untreated whole blood stimulated by *L. infantum* promastigotes; Neu+LXA4, untreated neutrophils stimulated by LXA4; Neu+LXA4+Leish, LXA4-pretreated neutrophils stimulated by promastigotes; Neu+Boc+LXA4, Boc-pretreated neutrophils stimulated by LXA4

Given the prominent role of the lipoxin A4 receptor in the anti-inflammatory response of neutrophils, we explored its possible role in the release of citrullinated histone H3 (H3Cit, a significant biomarker of NETs) by using flow cytometric analysis. The thresholds were determined based on the fluorescence levels of the isotype controls. The neutrophils that were positive for H3Cit were counted. As shown in Fig. 2, there were significantly differences among experimental groups (*F*_(5,12)_ = 2.285, *P* < 0.001). The proportion of H3Cit^+^ neutrophils in the Boc-primed whole blood samples as compared to the unprimed whole blood samples on stimulation with promastigotes was significantly decreased (*P* = 0.002) (Fig. 2b, c). Despite being less effective than the promastigotes, lipoxin A4 was found to increase H3Cit extrusion significantly as compared to control group (*P* = 0.003) (Fig. 2a, e). Furthermore, Boc was found to reverse the agonistic effects of LXA4 (*P* = 0.003) (Fig. 2e, g).

**Fig. 3:**
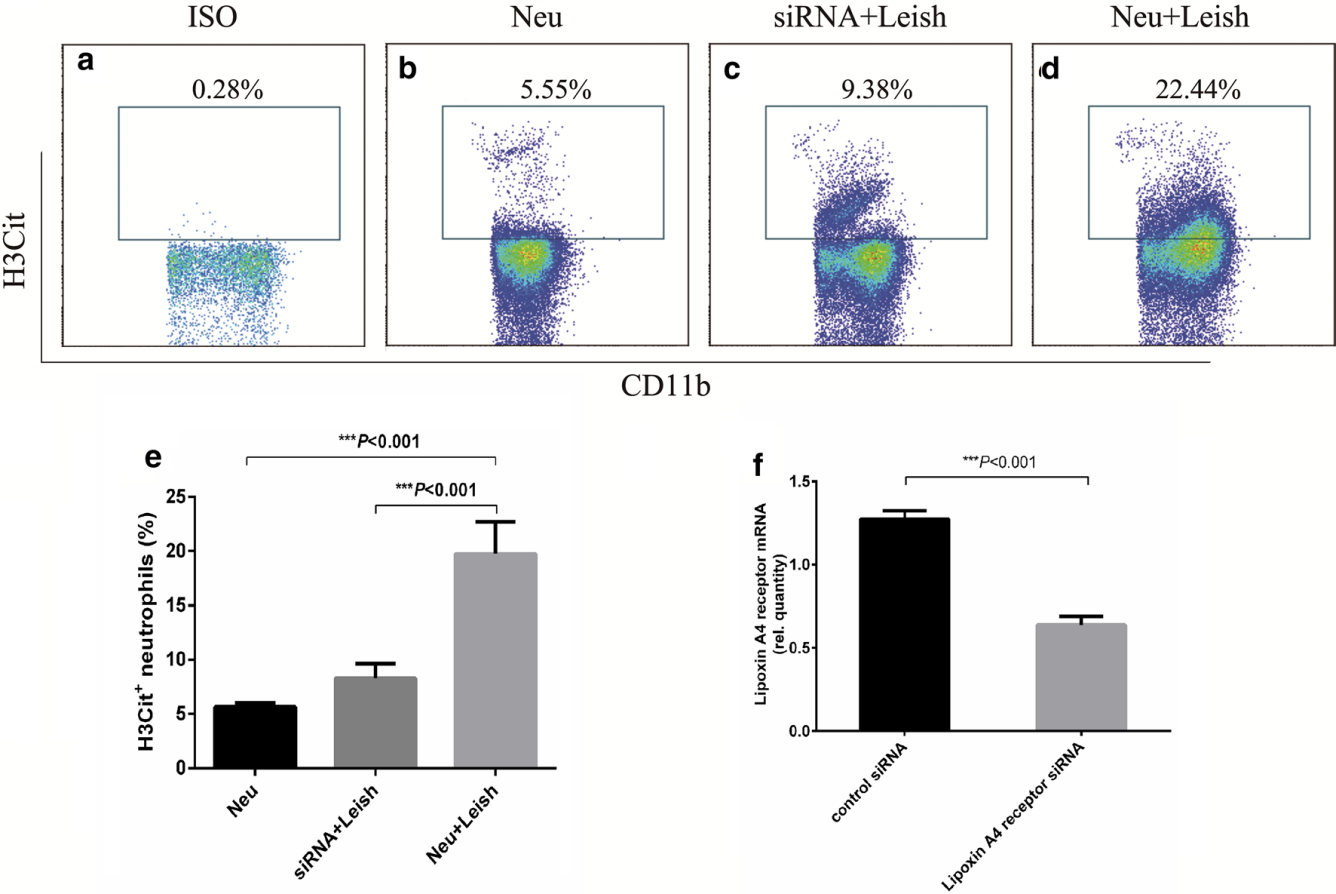
H3Cit formation in neutrophil-like HL-60 cells with/without lipoxin A4 receptor silencing after stimulation by *L. infantum* promastigotes for 5 h. HL-60 cells induced to differentiate into neutrophil-like cells (with 1.25% DMSO) with or without lipoxin A4 receptor gene silencing were exposed to *L. infantum* promastigotes or left untreated for 5 h at 37 °C. H3Cit formation was quantified by flow cytometric assay. **a** Isotype of H3Cit. **b** Percentage of H3Cit-positive differentiated HL-60 cells in experimental group Neu; **c** Percentage of H3Cit-positive differentiated HL-60 cells in experimental group siRNA+Leish; **d** Percentage of H3Cit-positive differentiated HL-60 cells in experimental group Neu+Leish; **e** Comparison of the H3Cit-positive neutrophil percentages among experimental groups. The differences were analyzed by one-way ANOVA, then LSD was applied in the following two groups’ comparison; **f** HL-60 cells were transfected with control siRNA or lipoxin A4 receptor-specific siRNA. Lipoxin A4 receptor transcripts were quantified relative to GAPDH mRNA, using qRT-PCR. Differences were analyzed by Student’s *t*-test. *P < 0.05, **P < 0.01, ***P < 0.001. *Abbreviations*: Neu, differentiated HL-60 cells without treatment; siRNA+Leish, differentiated HL-60 cells in lipoxin A4 receptor-silenced HL-60 cells exposed to *L. infantum* promastigotes; Neu+Leish, differentiated HL-60 cells stimulated by *L. infantum* promastigotes

As neutrophils have a short lifespan after isolation, HL-60 cells that can differentiate into neutrophil-like cells in response to 1.25% DMSO were used as a model [21] to investigate the impact of the lipoxin A4 receptor on NET expression. We induced the differentiation of HL-60 cells into neutrophil-like cells and the silencing of the lipoxin A4 receptor in neutrophil-like HL-60 cells (*t*_(4)_ =15.378, *P* < 0.001) (Fig. 3f), and then determined *Leishmania*-induced H3Cit production by flow cytometric analysis in both the silenced and non-silenced cells. As shown in Fig. 3, there were significantly differences among experimental groups (*F*_(2,6)_ = 3.264, *P* < 0.001). The proportion of H3Cit^+^ neutrophils in the lipoxin A4 receptor-silenced HL-60 samples as compared to the unsilenced HL-60 samples stimulated by promastigotes was significantly decreased (*P* < 0.001) (Fig. 3c, d). Our data thus suggest that the lipoxin A4 receptor mediates *Leishmania*-associated NETosis.

### The lipoxin A4 receptor regulates the release of NETs during *L. infantum* stimulation

**Fig. 4:**
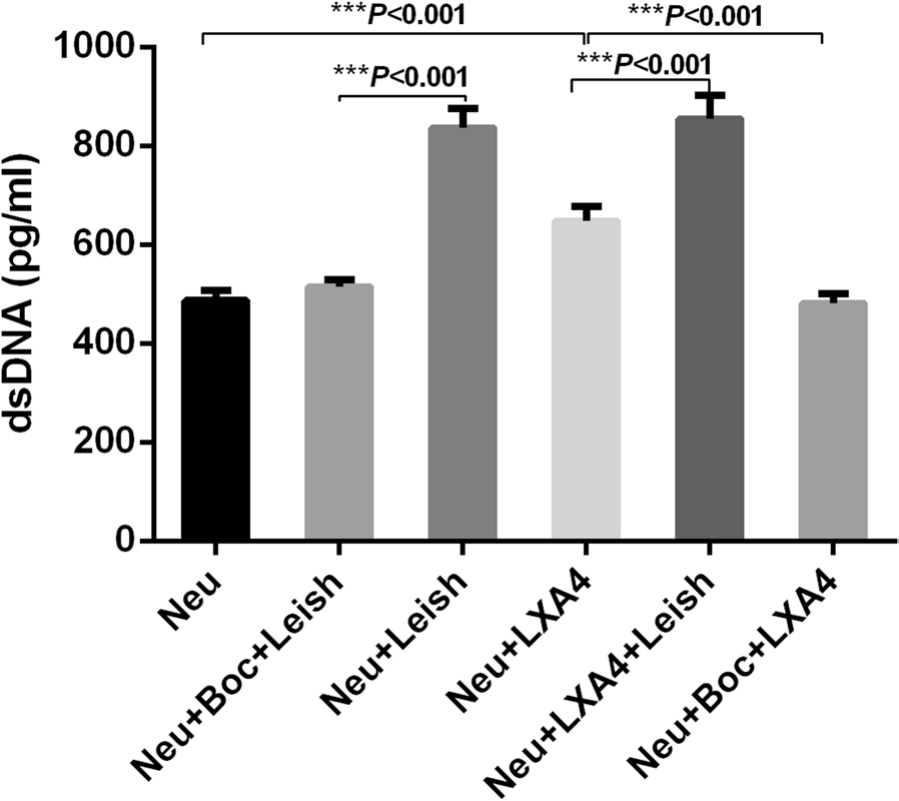
Comparison of extracellular dsDNA among the experimental groups. Purified neutrophils pre-treated with or without the antagonist (Boc) or the agonist (LXA4) were exposed to *L. infantum* promastigotes for 5 h at 37 °C. Extracellular dsDNA in the culture supernatant of the experimental groups were quantified and compared to assess the NETs production. The differences were analyzed using one-way ANOVA, then LSD was applied in the following two groups’ comparison. *P < 0.05, **P < 0.01, ***P < 0.001. *Abbreviations*: Neu: control group of untreated neutrophils; Neu+Boc+Leish, Boc-pretreated neutrophils stimulated by *L. infantum* promastigotes; Neu+Leish, untreated neutrophils stimulated by *L. infantum* promastigotes; Neu+LXA4, untreated neutrophils stimulated by LXA4; Neu+LXA4+Leish, LXA4-pretreated neutrophils stimulated by promastigotes; Neu+Boc+LXA4, Boc-pretreated neutrophils stimulated by LXA4

**Fig. 5:**
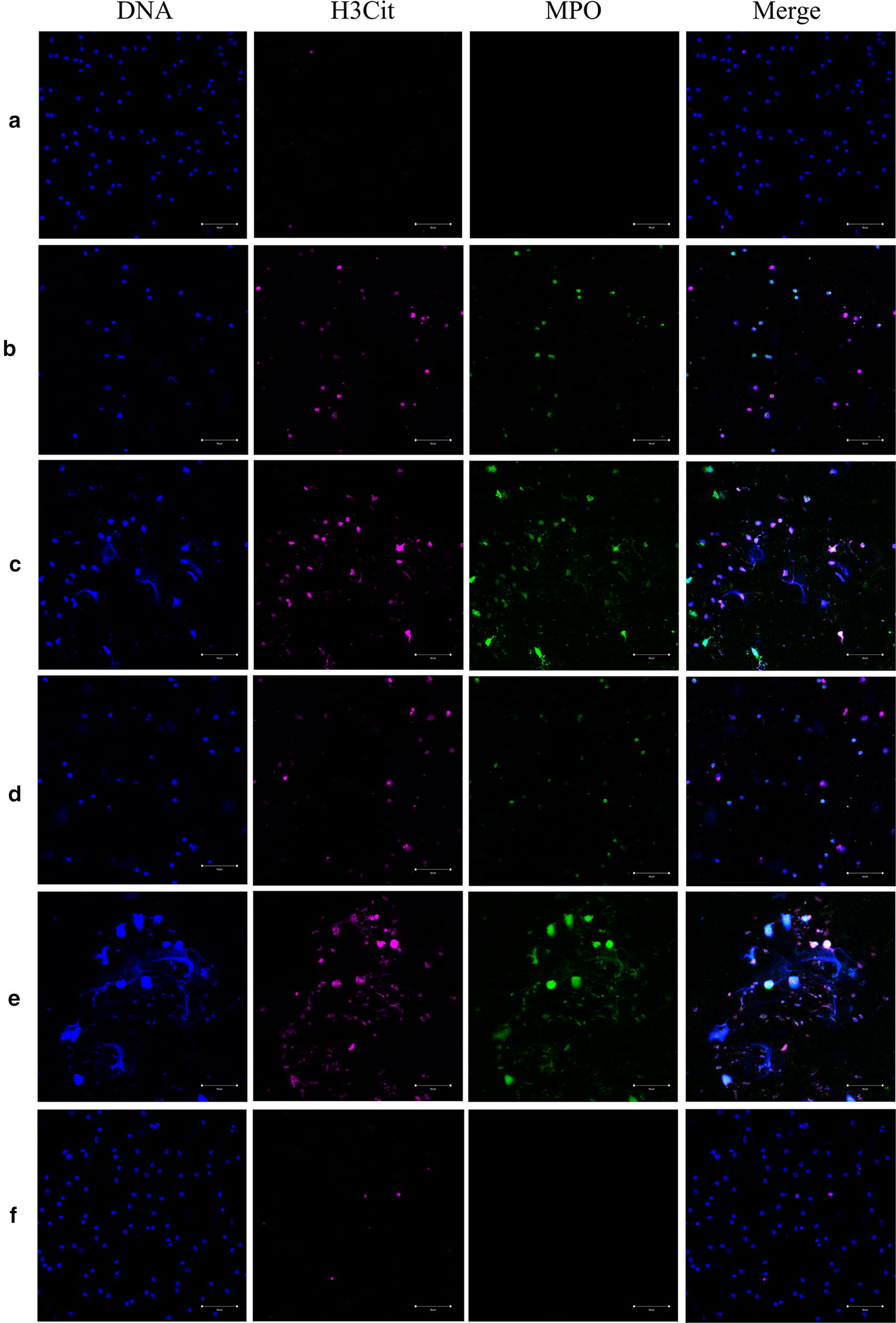
Visualization of NETs by fluorescence staining of DNA, H3Cit and MPO. Purified peripheral blood neutrophils pre-treated with or without the antagonist (Boc) or the agonist (LXA4) were exposed to *L. infantum* promastigotes for 5 h at 37 °C and immunostained for DNA (blue), H3Cit (red) and MPO (green). DNA: DNA backbone is stained with DAPI. H3Cit: citrullinated histone H3 is visualized with a red fluorescence label. MPO: myeloperoxidase is detected with a green fluorescence-labeled specific antibody. Merge: merged image showing fluorescence staining of DNA, H3Cit and MPO. **a** Control group of untreated neutrophils; **b** Boc-pretreated neutrophils stimulated by *L. infantum* promastigotes; **c** Untreated neutrophils stimulated by *L. infantum* promastigotes obviously showing DNA net-like fibers and releasing H3Cit and MPO; **d** Untreated neutrophils stimulated by LXA4 expressing H3Cit and MPO; **e** LXA4-pretreated neutrophils stimulated by promastigotes obviously exhibiting DNA net-like fibers and releasing H3Cit and MPO; **f** Boc-pretreated neutrophils stimulated by LXA4 showing intact cell structure. NETs positive for DNA, H3Cit and MPO were visible in Boc-untreated and lipoxin A4 pre-treated neutrophils stimulated by promastigotes. However, most cells in the Boc-primed neutrophil samples showed intact and smaller nuclei; this means that Boc had an inhibitory effect on NET extrusion *via* its antagonistic effects on the lipoxin A4 receptor. *Scale-bars*: 100 µm

As a significant biomarker of NETs, the antagonistic effect of Boc on H3Cit production implies that the lipoxin A4 receptor may have an effect on NET extrusion. Purified neutrophils primed by an antagonist or agonist were stimulated with promastigotes. Our extrapolation was confirmed by the dsDNA evaluation (Fig. 4) and fluorescence microscopy findings (Fig. 5).

As shown in Fig. 4, there were significantly differences among experimental groups (*F*_(5,12)_ = 1.919, *P* < 0.001). The dsDNA concentration in the Boc-primed neutrophil samples as compared to the unprimed neutrophil samples stimulated by promastigotes was significantly decreased (*P* < 0.001), and LXA4 exhibited an agonistic effect on the dsDNA concentration as compared to the control samples (*P* < 0.001).

Similarly, the fluorescence microscopy results also showed that NETs positive for DNA, H3Cit and MPO were visible in Boc-untreated and lipoxin A4 pre-treated neutrophils stimulated by promastigotes (Fig. 5c, e). However, most cells in the Boc-primed neutrophil samples showed intact and smaller nuclei (Fig. 5b, f), which means that Boc had an inhibitory effect on NET extrusion *via* its antagonistic effect on the lipoxin A4 receptor.

## Discussion

In the present study, we tried to explore whether the lipoxin A4 receptor plays a role in *L. infantum*-induced NETs formation. Our findings indicate that NETs positive for DNA, H3Cit and MPO were visible in *Leishmania*-infected neutrophils purified from mice. However, most of the lipoxin A4 receptor-blocked neutrophils showed intact and smaller nuclear morphology without MPO fluorescence. Moreover, Boc can reverse the effect of the agonist and downregulate NET extrusion. These observations were quantitatively confirmed by dsDNA measurement. Our research explored the possible role of the lipoxin A4 receptor as a trigger for the release of NETs, and findings confirmed the possibility of such a function. A more important finding is that *Leishmania* stimulation can significantly increase the NET extrusion of lipoxin A4-primed neutrophils, even though lipoxin A4 could stimulate the NET extrusion. This finding suggest that the lipoxin A4 receptor is one of the receptors that mediates *L. infantum*-induced NET formation, which may rely on a complex regulatory process.

H3Cit plays a crucial role in immune cell chromatin decondensation, as a result of which it can be detected in the nuclei of neutrophils upon stimulation [22, 23]; furthermore, H3Cit has been proposed to be a central marker of NETs [24]. As flow cytometric assays for direct quantification of NET markers of whole blood [25] can eliminate the effect of isolation procedures on neutrophils, whole blood was used to study the role of the lipoxin A4 receptor in the production of NETs by flow cytometry analysis of specific cell populations without purification. Our research found that the quantity of neutrophils positive for H3Cit is synchronously decreased in neutrophils that are pre-blocked with the lipoxin A4 receptor antagonist Boc and is reversely increased by lipoxin A4 activation before stimulation with promastigotes. Further confirmation is that there was a significant decrease in the proportion of H3Cit-positive neutrophils in lipoxin A4 receptor-silenced HL-60 cells compared with non-silenced neutrophil samples stimulated by promastigotes. The H3Cit expression is consistent with the confocal observations and dsDNA measurement. Research has demonstrated that the proportion of neutrophils positive for intracellular H3Cit [26] and the presence of plasma H3Cit [27] is correlated with disease severity, and that H3Cit can serve as a potential biomarker and even therapeutic target of disease [27].

Apart from their ability to regulate neutrophil activation and recruitment, these ligands have a profound influence on neutrophil survival and apoptosis with contrasting effects [19]. Lipoxin A4 is considered as an important anti-inflammatory lipid mediator that is well known as the “stop signal” in inflammatory reactions [28]. Therefore, *Leishmania* promastigotes may inhibit inflammatory reactions by activating the lipoxin A4 receptor and thereby inducing NETosis. With the establishment of the concept of NET anomaly and exploration of the pathological mechanism of NETs, a better understanding of the signal pathway of NETosis would be of great help to launch interventions. The activation of ERK downstream of PI3Kγ, which occurs *via* reactive oxygen species-dependent pathways, is associated with *L. amazonensis*-induced NETosis [29]. However, the pathways that are present downstream of *Leishmania*-induced NET require further exploration. Research has shown that the lipoxin A4 receptor slows down disease progression by inhibiting inflammatory responses and the activation of NF-κB and/or MAPK pathways [30, 31]. With regard to p38 MAPK and NF-κB pathways, research has reported that they are required for NET formation [32]. We speculated that the lipoxin A4 receptor may mediate the progression of NETs *via* NF-κB and ERK/p38 MAPK-dependent pathways in *L. infantum* infection. Further experiments to investigate the participation of lipoxin A4 receptor signaling in *L. infantum*-induced NET formation are needed.

Can NETs effectively resist *Leishmania* invasion? A study showed that *L. amazonensis* promastigotes were damaged after being captured by NETs, which confirmed the killing effect of NETs *in vitro* [11]. However, NETs failed to kill *L. mexicana in vivo* [9]. Based on the DNA skeleton structure of NETs, NETs can be degraded by nucleases. The *Leishmania* membrane anchors a class I nuclease, 3-nucleotidase/nuclease [33], which can destroy the structure of NETs and help the protozoa escape the immunity of NETs [12]. Therefore, whether *Leishmania* is effectively cleared by NETs is affected by many factors, such as the particular protozoan strain and host characteristics. The desert sub-type strain (MHOM/CN/08/JIASHI-5) involved in this study, which causes the majority of cases (93.70%) occurring in the zero- to two-years age group, is highly pathogenic and endemic in the northwestern desert regions of China [34]. Interestingly, comparing the protein expression of *L. infantum* strains from different regions of China by using non-labeled quantitative proteomics, we found that the desert sub-type strain significantly overexpressed 3-nucleotidase/nuclease [35]. Therefore, the desert sub-type strain could effectively be protected against NETs. We speculate that there may be some correlation with the characteristics of the desert sub-type of zoonotic visceral leishmaniasis, but this requires further demonstration.

The overexpression of NETs may have detrimental effects [36], while constitutive activation, dysregulation of suppressive mechanisms and excess NET yield are prominent pathogenic mechanisms that are also likely to contribute to the disease. That is, they could have beneficial or detrimental effects that vary by context. As neutrophils release NETs in response to *Leishmania* species, it has been proposed that NETs play distinct roles according to the progression of the infection [37, 38]. The ability of NETs to damage epithelial and endothelial cells and hepatic tissues is well documented [39, 40]. There is probably an optimal level of NETs that needs to be explored, for which a better understanding of the functions and impact of NETs on health would be useful. DNase treatment was used to abrogate NET-mediated chronic inflammation [41]. Further investigation to evaluate the role of the lipoxin A4 receptor in promoting or controlling infection with *Leishmania in vivo* is the next-step research focus. The lipoxin A4 receptor for neutrophil-mediated immune response to *L. infantum* infection in our study might offer an alternative target for NET control.

## Conclusions

This study assessed the role of the lipoxin A4 receptor in the neutrophil-mediated immune response to *L. infantum* infection. A better understanding of the immunological mechanism of NETs in the context of health would be helpful in terms of using this receptor for targeted treatment.

## Methods

### Parasite and mice

The *L. infantum* isolate (MHOM/CN/08/JIASHI-5) was supplied by Dr. Wang (NIPD, China CDC) and was maintained in the promastigote stage at 26 °C by passage in 199 medium (Lonza Group, Basel, Switzerland) supplemented with heat-inactivated fetal bovine serum (FBS; 30 min, 56 °C; Sera Laboratories International, Horsted Keynes, UK) and 100 U/ml penicillin + 100 μg/ml streptomycin (BioWhittaker, Verviers, Belgium). Parasite concentration was assessed in a Neubauer counting chamber (Paul Marienfeld GmbH & Co. KG, BM, Germany).

Five-week-old female BALB/c mice (body weight: 16 ± 2 g) were purchased from Shanghai Jihui Laboratory Animal Feeding Co. Ltd. The mice were adaptively fed with standard laboratory chow and sacrificed for research. All experimental procedures were approved by the Laboratory Animals Welfare and Ethics Committee of National Institute of Parasitic Diseases, Chinese Center for Diseases Control and Prevention.

### *In vitro L. infantum* infection and flow cytometric analysis of whole blood

Whole blood samples were obtained through the retro-orbital bleeding method and collected in tubes containing heparin and used within 1 h. A 0.2-ml volume of heparin-containing blood from each mouse was seeded in a 24-well plate containing *L. infantum* promastigotes at a parasite:neutrophil ratio of 10:1 in 300 μl of RPMI 1640 medium supplemented with 10% heat-inactivated FBS (v/v) for 5 h. There were six experimental groups: whole blood stimulated by promastigotes without pretreatment, whole blood pre-incubated for 30 min with 10 μM Boc prior to stimulation with promastigotes or 10 ng/ml LXA4 (an endogenous lipid mediator/activator of the lipoxin A4 receptor), whole blood pre-treated with 10 ng/ml LXA4 with or without promastigote stimulation later, and whole blood without any treatment was used as the control group. Three independent experiments were performed. The NETs were quantified by flow cytometric assay.

### Isolation of neutrophils from peripheral blood

Whole blood samples from mice were obtained through the retro-orbital bleeding method, collected in tubes containing heparin and used within 1 h. Neutrophil isolation was achieved using mouse peripheral blood neutrophil purification kit (TBD, Nanjing, China). Isolated cells were suspended in RPIM 1640 supplemented with 10% heat-inactivated FBS (v/v) (Gibco, San Diego, CA, USA), and their viability and concentration were assessed by trypan blue exclusion in a Neubauer counting chamber before any of the assays were performed. Cell purity was determined by flow cytometry analysis and microscopic observation. Neutrophil purity was confirmed to be > 85% by immunostaining with CD11b and Ly6G.

### *In vitro* stimulation of purified neutrophils with *L. infantum* promastigotes

Purified neutrophils (final concentration, 2 × 10^5^ cells/well) were seeded in 24-well plates (Nunc, Roskilde, Denmark) with *L. infantum* promastigotes at a parasite-neutrophil ratio of 10:1 in RIPM 1640 supplemented with 10% heat-inactivated FBS (v/v). Plates were incubated at 37 °C in a humidified atmosphere containing 5% of CO_2_ for 5 h to allow the induction of NETs. The experimental groups were the same as described earlier. The neutrophil cultures were used in the following experiments: measurement of extracellular dsDNA and microscopic assessment of NETs.

### HL-60 cell culture

The human acute promyelocytic leukemia cell line HL-60 (provided by the Cell Bank of the Chinese Academy of Sciences) was maintained in RIPM 1640 medium supplemented with 10% heat-inactivated FBS (v/v) in 5% CO_2_ at 37 °C. Cells were seeded at a density of 5 × 10^5^ cells/ml and cultured for 5 days in the above-mentioned cell medium supplemented with 1.25% DMSO to induce their differentiation into neutrophil-like cells [21]. Fresh medium was added on the third day of culture to prevent cell overgrowth and depletion of nutrients.

### Lipoxin A4 receptor gene silencing

HL-60 cells stimulated to undergo neutrophil-like differentiation were seeded in 24-well plates (final concentration, 2 × 10^5^ cells/well). On day 3 of differentiation, the cells were transfected with control or specific siRNA against the lipoxin A4 receptor [42] by using Lipofectamine™ RNAiMAX (Thermo Fisher Scientific, Waltham, MA, USA) for 48 h Then, 3 μl of the transfection reagent and 50 nM siRNA were diluted separately in 50 μl of Opti-MEM (Gibco) and incubated for 10 min at room temperature. The above solutions were mixed and incubated for a further 5 min. Subsequently, the Lipofectamine-siRNA complexes (50 μl) were added to the culture medium and the cells were incubated for 48 h for further analysis.

### Quantitative reverse transcriptase-polymerase chain reaction

The total RNA of HL-60 cells was extracted with Trizol reagent (Invitrogen, Carlsbad, CA, USA) and reverse-transcribed using a cDNA reverse transcription kit (TaKaRa, Dalian, Japan). To determine the efficiency of lipoxin A4 receptor gene down-regulation, the reverse-transcribed cDNA was used as a template in qPCR reaction mixtures containing SYBR Green Real-time PCR Master Mix (TaKaRa) and 0.4 μM forward and reverse primers. The following primers were utilized: GAPDH forward [21] (5′-CCC CTT CAT TGA CCT CAA CTA C-3′), GAPDH reverse (5′-GAG TCC TTC CAC GAT ACC AAA G-3′); lipoxin A4 receptor forward [43] (5′-GTG ATC TGG GTG GCT GGA TT-3′); and lipoxin A4 receptor reverse (5′-AGG GCC AGG TTC AGG TAA CA-3′). Data are shown as relative mRNA levels normalized to GAPDH.

### *In vitro* stimulation of differentiated HL-60 cells with *L. infantum* promastigotes

Differentiated HL-60 cells with or without lipoxin A4 receptor gene silencing (final concentration, 2 × 10^5^ cells/well) were seeded in 24-well plates (Nunc) with *L. infantum* promastigotes at a parasite-cell ratio of 10:1 in RIPM 1640 medium supplemented with 10% heat-inactivated FBS (v/v). Plates were incubated at 37 °C in a humidified atmosphere containing 5% of CO_2_ for 5 h to allow the induction of NETs. Differentiated HL-60 cells without any treatment was used as the control group. Three independent experiments were performed. NET quantification was performed with flow cytometric analysis.

### Flow cytometric analysis of NETs

The *in vitro* infected samples were collected into tubes and centrifuged at 1000× *g* for 10 min. The precipitate was incubated with 2 μl of rabbit polyclonal anti-Histone H3 antibody (citrulline R2 + R8 + R17) (Abcam, Cambridge, UK) for 30 min at room temperature. Following this, 1 μl of anti-CD11b-PE (eBioscience, San Diego, CA, USA) and anti-Ly6G-BV421 (eBioscience) antibodies, and 0.8 μl of AlexaFluor-700-conjugated goat anti-rabbit antibody (Thermo Scientific, Rockford, IL, USA) were added and incubated for 30 min at room temperature in the dark. Then, for whole blood samples, 1 ml of FACS reagent (BD Biosciences, San Diego, CA, USA) was added until red cell lysis was achieved (10−15 min). This was followed by the addition of 1 ml of 2% bovine serum albumin in phosphate-buffered saline (PBS) and centrifugation at 1000× *g* for 10 min. The supernatant was discarded, and the pellet was resuspended in 300 μl of PBS. Labeled cells were kept on ice and examined with flow cytometry analysis (FACS LX, Beckman-Coulter, Pasadema, CA, USA), which was performed with the CytExpert software (Beckman). The percentage of isotype controls was < 3%. At least 10,000 neutrophil or neutrophil-like populations were collected for each sample. Three independent experiments were performed.

### Measurement of extracellular dsDNA

NETs were quantified in the culture supernatant with the Quant-iT™ PicoGreen® dsDNA Assay kit (Thermo Fisher Scientific) according to the manufacturer’s instructions. Samples were distributed into 96-well plates and read in a spectrofluorometer (SpectraMax M5; Molecular Devices, Sunnyvale, CA, USA) with a filter setting of 480 nm (excitation) and 520 nm (emission).

### Microscopic assessment of NETs

Fluorescence microscopy and scanning electron microscopy were used for the visualization of NETs. For fluorescence microscopy, slides with adherent neutrophils were fixed with 4% paraformaldehyde (Sigma-Aldrich, Santa Clara, CA, USA) for 20 min, blocked with mouse serum, stained with rabbit polyclonal anti-Histone H3 antibody (citrulline R2 + R8 + R17) (Abcam) at 4 °C overnight, and washed with PBS three times. This was followed by staining with AlexaFluor-700-conjugated goat anti-rabbit antibody (Thermo Scientific) and anti-MPO-FITC antibody (Abcam) for 60 min each. Finally, DAPI (4,6-diamidino-2-phenylindole; Sigma-Aldrich) staining was performed for 10 min. All specimens were observed, and photographs of the relevant fields were taken under a confocal microscope (20× objective, 10× eyepiece; ECLIPSE-TI, Nikon, Tokyo, Japan).

For scanning electron microscopy, the coverslips seeded with samples were washed with PBS, fixed with 1 ml Monti-Graziadei solution, and processed for scanning electron microscopy.

### Statistical analysis

Data were expressed as mean ± standard deviation (SD) of triplicate wells in three independent experiments. All statistical analyses were performed using SPSS version 20.0. Differences based on the data characteristics were analyzed by one-way ANOVA and Student’s t-test. All graphs were performed with the GraphPad Prism 7 software. *P*-values < 0.05 were considered to indicate statistical significance.

## Data Availability

The datasets supporting the conclusions of this article are included within the article.

## Abbreviations

NETs: neutrophil extracellular traps
Boc: *N-t*-Butoxycarbonyl-phenylalanine-leucyl-phenylalanine-leucyl-phenylalanine
LXA4: lipoxin A4
MPO: myeloperoxidase
H3Cit: citrullinated histone H3
